# A Low Dose of Fermented Soy Germ Alleviates Gut Barrier Injury, Hyperalgesia and Faecal Protease Activity in a Rat Model of Inflammatory Bowel Disease

**DOI:** 10.1371/journal.pone.0049547

**Published:** 2012-11-14

**Authors:** Lara Moussa, Valérie Bézirard, Christel Salvador-Cartier, Valérie Bacquié, Corinne Lencina, Mathilde Lévêque, Viorica Braniste, Sandrine Ménard, Vassilia Théodorou, Eric Houdeau

**Affiliations:** 1 Neuro-Gastroenterology and Nutrition, Institut National de la Recherche Agronomique, UMR1331 Toxalim, INRA/INPT/UPS, Toulouse, France; 2 GENIBIO, Lorp-Sentaraille, France; Institut Pasteur de Lille, France

## Abstract

Pro-inflammatory cytokines like macrophage migration inhibitory factor (MIF), IL-1β and TNF-α predominate in inflammatory bowel diseases (IBD) and TNBS colitis. Increased levels of serine proteases activating protease-activated receptor 2 (PAR-2) are found in the lumen and colonic tissue of IBD patients. PAR-2 activity and pro-inflammatory cytokines impair epithelial barrier, facilitating the uptake of luminal aggressors that perpetuate inflammation and visceral pain. Soy extracts contain phytoestrogens (isoflavones) and serine protease inhibitors namely Bowman-Birk Inhibitors (BBI). Since estrogens exhibit anti-inflammatory and epithelial barrier enhancing properties, and that a BBI concentrate improves ulcerative colitis, we aimed to evaluate if a fermented soy germ extract (FSG) with standardized isoflavone profile and stable BBI content exert cumulative or synergistic protection based on protease inhibition and estrogen receptor (ER)-ligand activity in colitic rats. Female rats received orally for 15 d either vehicle or FSG with or without an ER antagonist ICI 182.780 before TNBS intracolonic instillation. Macroscopic and microscopic damages, myeloperoxidase activity, cytokine levels, intestinal paracellular permeability, visceral sensitivity, faecal proteolytic activity and PAR-2 expression were assessed 24 h, 3 d and 5 d post-TNBS. FSG treatment improved the severity of colitis, by decreasing the TNBS-induced rise in gut permeability, visceral sensitivity, faecal proteolytic activity and PAR-2 expression at all post-TNBS points. All FSG effects were reversed by the ICI 182.780 except the decrease in faecal proteolytic activity and PAR-2 expression. In conclusion, the anti-inflammatory properties of FSG treatment result from two distinct but synergic pathways i.e an ER-ligand and a PAR-2 mediated pathway, providing rationale for potential use as adjuvant therapy in IBD.

## Introduction

Inflammatory bowel diseases (IBD), namely Crohn's disease (CD) and ulcerative colitis (UC), are chronic and relapsing inflammatory conditions characterized by an abnormal immune response to microbiota, impaired epithelial barrier function, tissue damage, and abdominal pain [1], [2], [3]. In both disorders, mucosal immune cells produce large amounts of chemokines and cytokines, including macrophage migration inhibitory factor (MIF), IL-1β and TNF-α, both orchestrating the immuno-inflammatory process leading to epithelial barrier defect, tissue damage [3]. On the other hand, proteases originated from various cell populations such as tryptase, trypsin, thrombin and cathepsin G may act as inflammatory mediators [4], [5] by cleaving and activating protease-activated receptors (PARs) which represent novel members of the G-protein-coupled receptor family. Among the PARs family, PAR-2 has been largely studied in the context of inflammation. Interestingly, the levels of potential activators of PAR-2 such as serine-proteases are increased in the colonic tissue of IBD patients [4], [5], [6]. Both PAR-2 activity and proinflammatory cytokines impair epithelial barrier by decreasing tight junction (TJ) protein expression [3], [4], hence facilitate the entry of luminal aggressors perpetuating inflammation and pain [7].

A variety of medical therapies have been used for treatment of IBD patients. Among them immunosuppressive medications are prominent [8], with benefits observed in both clinical remission and mucosal healing. However, beside these conventional therapies, patients with IBD often question clinicians about dietary suggestions to improve their symptoms and quality of life [9]. During the last decade, soy extracts have attracted attention because of their anti-inflammatory properties in animal models of IBD [10] partly due to a reduced expression of inflammatory mediators [11]. Soy extracts are mainly characterized by the presence of isoflavones and their content of a family of serine protease (trypsin-like) inhibitors known as Bowman-Birk Inhibitors (BBI) [12]. However, the isoflavone profile and concentration as well as the BBI content vary according to the extract considered. A beneficial effect in terms of rates of remission and clinical response has been observed in patients with UC treated with a concentrate of BBI [13]. Soy isoflavones such as genistein, daidzein and its metabolite equol exhibit estrogen-like activity [14]. In the colon, estrogen receptors (ERs) signalling enhance expression of transmembrane TJ proteins in non-inflamed conditions [15], and decrease proinflammatory cytokine production in experimental colitis [16], [17]. Soy isoflavones have been shown to enhance intestinal tight junction (TJ) barrier integrity [18] although the precise mechanism underlying amelioration of TJ barrier remains unclear. If recent attention has been made on the beneficial properties of purified genistein or isoflavone-enriched diet in colitis [10], [19], the estrogenic potential of isoflavones to enhance TJ barrier integrity in inflamed tissues remains to be investigated. This study was conducted using a fermented soy-germ (FSG) extract containing daidzein, glycitein and genistein present in aglycone forms resulting from fermentation [20], which represent the gut absorbable isoflavone forms [21], as well as stable levels of BBI.

Regarding the composition of the FSG extract used in this study, we aimed to evaluate whether this compound may exert cumulative or synergistic protection based on protease inhibition and ER-ligand activity in a model of experimental colitis in rats.

## Materials and Methods

### Ethics statement

All experimental protocols were approved by the Local Animal Care and Use Committee of Institut National de la Recherche Agronomique.

### Animals and sexual cycle stage determination

Female Wistar rats (Janvier SA, Le Genest St Isle, France) were housed under controlled conditions of temperature (21±1°C) and illumination (12 h light, 12 h dark) with free access to water and fed standard pellets (UAR pellets, Epinay, France). Estrous cycle stages were assessed through vaginal smears as previously described [15].

### Fermented soy germ ingredient

The FSG was industrially processed as ground powder under the trademark Primasoy® by GENIBIO (Lorp-Sentaraille, France). This compound had an average content of isoflavones of 34.7 µmol/g of product (55% daidzein, 30% glycitein and 15% genistein in aglycone forms). Bowman-Birk protease inhibitors were quantified in chymotrypsin inhibitory (CI) units, where 1 CI is the amount needed to inhibit 1 mg of bovine pancreatic chymotrypsin [20].

FSG was diluted in water and prepared daily in order to administer in 1 ml volume, 0.45 mg isoflavone aglycone forms equivalent/d/rat and 1 BBI CI/d/rat as described previously [22].

### Induction of experimental colitis

Colitis was induced by an intra-colonic (IC) administration of TNBS (2,4,6-trinitrobenzene sulphonic acid) at a dose of 80 mg/kg in 50% ethanol. TNBS was infused through a silicone catheter introduced 7 cm into the anus under acepromazine–ketamine anaesthesia, as previously described [23].

### Macroscopic damage scores

Immediately after sacrifice, the colon was removed and rinsed with saline. Intestinal damage was scored according to a modified scale of Wallace et al [24]. Briefly, the presence of mucosal hyperemia and bowel wall thickening, the severity and extent of ulceration and necrosis, the tissue adhesion, and the occurrence of diarrhea were rated according to a macroscopic damage score (MDS) ranging from 0 (normal appearance) to 13 (severe lesions).

### Microscopic damages

To evaluate microscopic damages (MD), samples of the distal colon were fixed in Duboscq-brazil solution during 24 h, dehydrated and embedded in paraffin. Samples were then cut into 5 µm thick transversal sections, mounted on glass slides, stained with hematoxylin and eosin, then observed with a light microscope (Nikon 90i, Nikon, France). The MD were assessed according to a modified histological grading scale described by Fabia and al [25]. Several parameters were observed as ulceration, mucus cell depletion, oedema, inflammatory cell infiltration and vessel dilatation. Each parameter estimated was graded 0–3 depending on the severity of the changes found: (0) no change, (1) mild, (2) moderated or (3) severe changes. MD were calculated by adding the scores of all parameters cited above.

### Myeloperoxidase activity assay

The activity of the enzyme myeloperoxidase (MPO), a marker of polymorphonuclear primary granules, was determined in the colon. Immediately after sacrifice, a distal colonic segment (1 cm long) was taken off, suspended in potassium phosphate buffer (KH_2_PO_4_ 44 mM, K_2_HPO_4_ 6 mM, pH 6.0), homogenized on ice with Polytron (PCU-2, Kinematica GmbH, Lucerne, Switzerland) and submitted to three cycles of freezing and thawing. Homogenates were then centrifuged at 10000 rpm for 15 min at 4°C. The pellets were resuspended in hexadecyl trimethylammonium bromide buffer (0.5% (wt/vol) in potassium phosphate buffer) to release MPO from polymorphonuclear neutrophil primary granules. These suspensions were sonicated (Büchi, Flawil, Switzerland) on ice and centrifuged at 10000 rpm for 15 min at 4°C. Supernatant fractions were diluted in potassium phosphate buffer containing 0.167 mg O-dianisidine dihydrochloride/ml and 0.00005% (vol/vol) H_2_O_2_. MPO from human neutrophils (Sigma, Saint Quentin Fallavier, France; 0.1 U/ml) was used as a standard. Changes in absorbance at 450 nm were recorded with a spectrophotometer (mc2UV, Safas, Monaco) every 10 s over 2 min. One unit of MPO activity was defined as the quantity of MPO degrading 1 µmol H_2_O_2_ min^−1^ ml^−1^ at 25°C. Protein concentrations (mg/ml) were determined using a modified method of Lowry (Detergent Compatible Assay, BioRad, Ivry/Seine, France) and MPO activity was expressed as MPO units/g protein.

### Tissue protein extraction and ELISA

Tissue proteins were extracted with RIPA buffer (1% Igepal, 0.5% deoxycholic acid, and 0.1% sodium dodecyl sulfate in Tris-buffered saline 1x; pH 7.4) with protease inhibitor cocktail (Roche Diagnostics, Mannheim, Germany). Clear lysates were prepared by centrifugation at 10000 g for 10 min, and protein concentrations were assessed using the BC Assay Uptima kit (Interchim). Samples were then processed for ELISA using commercial kits to determine colonic contents of IL-1β, and IL-10 (ELISA kits, Duoset R&D Systems, Lille, France). Data were expressed as concentration per mg of total proteins.

### MIF expression by Western blot

Briefly, tissue proteins were extracted with RIPA buffer as above, and equal protein amounts of each extract were separated in 15% SDS-polyacrylamide gel and transferred onto 0.45 µm nitrocellulose membranes (Whatman, Dominique Deutscher, Brumath, France). Membranes were blocked with Odyssey blocking buffer (Rockland, Tebu-bio, France), then incubated overnight at 4°C with the rabbit anti-MIF antibody (Torrey Pines Biolabs; 1/1000) or anti-GAPDH antibody (Cell signaling Technology, Ozyme, St Quentin-en Yvelines, France; 1/1000) used as internal standard. After washing, fluorescent CF770 anti-rabbit antibody (Biotium, Hayward, CA; 1/1000) was added for 1 hour at room temperature and protected from light. Membranes were scanned and the intensity of bands was analysed on infrared imaging system Odyssey (Li-Cor, Lincoln, NE). MIF expression was assessed relative to GAPDH for each sample analysed.

### Faecal proteolytic activity assay

Supernatants of faecal homogenates (25 µl) were incubated with 1 ml of reaction buffer (0.15 M/L NaCl and 20 mmol/L Tris-HCl, pH 8.5) and 1 ml of 0.5% (w/v) azocasein (Sigma, St.Quentin Fallavier, France) at 37°C. The reaction was stopped after 20 min with 1 ml of 10% (v/v) trichloroacetic acid (Sigma). Following centrifugation, absorption of the clear supernatant was measured at 366 nm. Proteolytic activity of the supernatants was normalized to protein content.

### PAR-2 immunohistochemistry

Colonic tissue samples were fixed in 4% formalin and incubated for 24 h in 30% of sucrose at 4°C. Samples were embedded in Tissue Tek medium (Euromedex, Souffelweyesheim, France) and frozen in isopentane at −45°C. Cryostat sections (7 µm) were post-fixed with acetone (10 min, −20°C) and hydrated in phosphate-buffered saline (PBS)-milk (0.1%). After incubation in blocking solution (PBS-0.25% Triton X100-0.1% BSA), sections were incubated overnight at 4°C with primary goat polyclonal antibody for PAR-2 (1/1000) (SantaCruz, California, USA). Sections were then washed in PBS-milk (0.1%) and incubated for 1 h at room temperature with biotinylated donkey anti-goat secondary antibody (1/1000) (Interchim, Montluçon, France), then 45 min at room temperature with FITC-avidine (1/500) (Clinisciences, Montrouge, France). Sections were mounted in Vectashield Hard set mounting medium (Clinisciences, Montrouge, France) and examined under a Nikon 90i fluorescence microscope (Nikon, Champigny-sur-Marne, France). PAR-2 area fraction per µm^2^ of epithelium was quantified using Nikon-Elements-Ar software.

### Intestinal paracellular permeability

Intestinal paracellular permeability (IPP) was performed using ^51^Cr-EDTA (Perkin–Elmer Life Sciences, Paris, France) as a marker of tight junctions paracellular permeation. ^51^Cr-EDTA (0.7 µCi) was diluted in 500 µl of saline and administered *per os* 24 h, 3 d or 5 d after induction of colitis. Animals were placed in metabolic cages, and faeces and urine were collected separately during 24 h. The radioactivity in urine was measured on a gamma counter (Cobra II, Packard Meriden, CT, USA). Permeability to ^51^Cr-EDTA was expressed as percentage of administered radioactivity recovered in 24 h urines.

### Surgical procedure

Animals were surgically prepared for abdominal striated muscle electromyography according to a previously described technique [23]. Briefly, rats were equipped with three groups of three NiCr wire electrodes (60 cm in length, 80 µm in diameter) implanted into the abdominal external oblique musculature. Electrodes were exteriorized on the back of the neck and protected by a glass tube attached to the skin.

### Distension procedure and electromyographic recordings

Rats were accustomed to be in polypropylene tunnels (6 cm diameter, 25 cm length) for 2 d before colorectal distension (CRD). A balloon (2 mm diameter; 4 cm length) consisting of a latex condom was introduced into the anus, fixed at the base of the tail and connected to a computerized barostat INRA [26]. The balloon was inflated progressively in steps of 15 mmHg, each step of inflation lasting 5 min. Colorectal pressure and balloon volume (referring to the intestinal compliance) were continuously monitored on a potentiometric recorder (L6517; Linseis, Selb, Germany) with a paper speed of 1 cm.min^−1^. The striated muscle spike bursts, related to abdominal cramps, were recorded on an electroencephalograph machine (Mini VIII; Alvar, Paris, France) from implanted electrodes. Differential amplification, using a short time constant (0.03s), allow us to detect high frequency spike bursts corresponding to abdominal cramps.

### Experimental protocol

Female Wistar rats were divided into 11 groups (n = 10 per group) and received orally for 15 d either vehicle (Ve; 1 ml of water) or FSG, with or without the ER antagonist ICI 182.780 (2 mg/kg/day, s.c) administered from day 11 until 15 of the treatment. Colitis was induced by IC instillation of TNBS/ethanol (80 mg/kg) on day 15 of the treatment. Non-inflamed rats received IC instillation of sterile saline solution. Body weight was evaluated every 2 days from the beginning of the treatment until 5 d post-TNBS. In a first series, animals were sacrificed 24 h, 3 d and 5 d post-colitis. MDS were determined at all time points post-TNBS. Colonic samples were taken for (i) MD and MPO activity assessments, (ii) IL-1β, IL-10 level measurements, (iii) MIF and PAR-2 expression. Faecal pellets were collected for proteolytic activity assessment. In a second series, IPP to oral ^51^Cr-EDTA was measured 24 h, 3 and 5 d post-TNBS in 24 h urines. Finally, in a last series at 5 d post-TNBS, visceral sensitivity in response to CRD was evaluated in animals previously equipped with NiCr electrodes implanted in the abdominal muscle.

Two additional sets of experiments were also conducted. In the first one, a group of 10 male Wistar rats received orally for 15 d the FSG treatment. On day 15, TNBS colitis was induced as described above, and MPO activity was evaluated 24 h and 3 d post-TNBS. In the second one, to test curative effects of FSG treatment, 10 female Wistar rats were orally given FSG starting the day of TNBS instillation until 5 days post-TNBS. MPO activity was evaluated on days 1, 3 and 5 post-TNBS.

### Statistical analysis

All data are presented as means±SEM. Statistical analysis were performed using Graph Pad Prism 4.0 (GraphPad, San Diego, CA). One-way ANOVA followed by Tukey's post-test was used to examine the effects of FSG treatment on TNBS-evoked IBD-like symptoms. Statistical significance was set at p<0.05.

## Results

### Effects of FSG on body weight

After 15 days of treatment with FSG, animals did not show any difference in body weight compared with control rats orally treated with Ve (data not shown). Body weight loss started 24 h after TNBS induction of colitis, to reach ∼13% on day 3 post-TNBS compared to non-inflamed rats, then started increasing (∼3%) 5 d after TNBS administration. Rats treated with FSG showed similar body weight loss after TNBS administration compared to Ve-treated colitic rats (Table 1).

**Table 1:** Body weight loss (g) in Ve or FSG-treated colitic rats.

Days post-TNBS	Ve-treated group	FSG-treated group
1	13±1.7	10±1.3
3	39±3.6**	35±2.7**
5	33±4.1*	29±3.5*

Values are presented as means ± SEM.

* p<0.05 *vs* body weight before TNBS administration,

** p<0.01 *vs* body weight before TNBS administration.

(One way-ANOVA followed by Tukey test).

### FSG treatment attenuates the severity of TNBS-induced colitis

**Figure 1:**
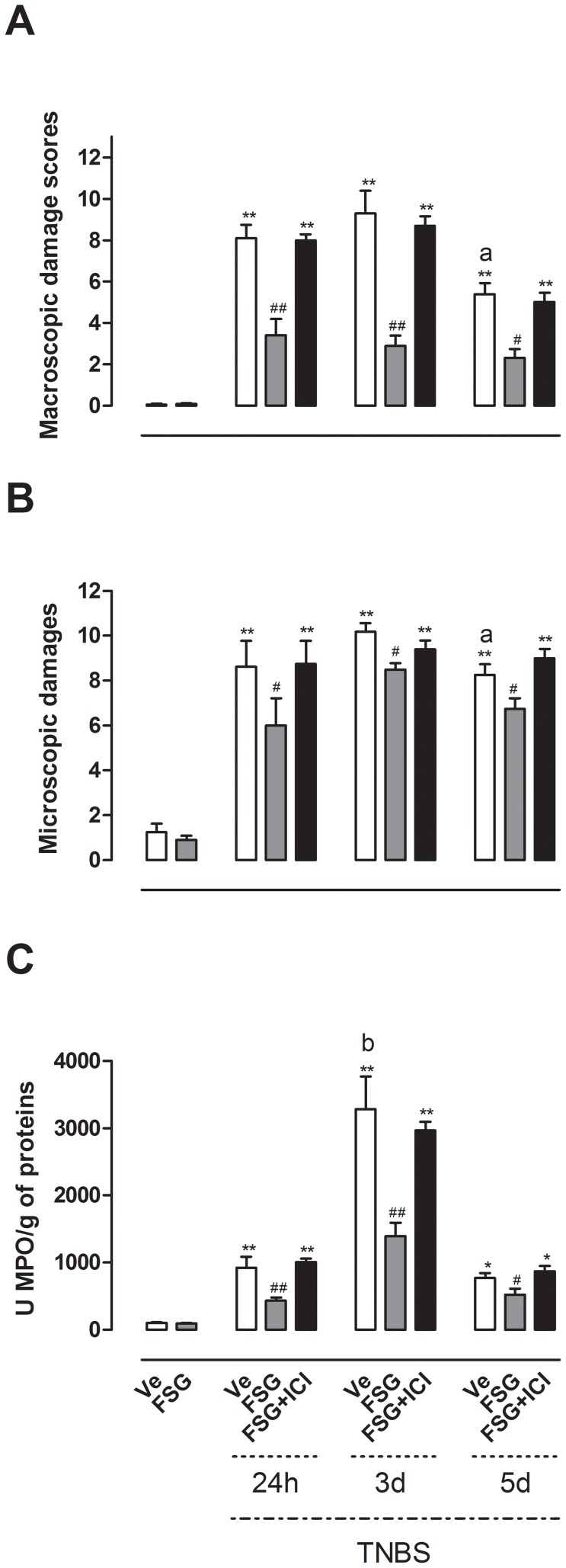
Effects of FSG treatment on the severity of TNBS-induced colitis. (A) MDS, (B) MD, (C) MPO activity were evaluated in presence or absence of the ER antagonist ICI 182.780. Values are presented as means ± SEM. Statistical analysis were performed by One way-ANOVA with post hoc comparison by Tukey test. *p<0.05 *vs* noninflamed group, **p<0.01 *vs* noninflamed group, ^#^p<0.05 *vs* inflamed vehicle, ^##^p<0.01 *vs* inflamed vehicle, ^a^p<0.05 *vs* Ve-treated rats 3 d post-TNBS, ^b^p<0.05 *vs* Ve-treated rats 24 h and 5 d post-TNBS.

TNBS administration resulted in colon inflammation associated with hyperemia, ulceration and bowel wall thickening, leading to a significant increase (p<0.01) in MDS 24 h, 3 d and 5 d after colitis induction compared with non colitic animals (Figure 1A). Oral FSG treatment significantly decreased MDS from days 1 to 5 after TNBS (p<0.05) (Figure 1A).

Microscopic damage evaluation by histology has shown that colitic rats exhibited disruption of the epithelial barrier, oedema, vessel dilatation and a marked infiltration of inflammatory cells on days 1, 3 and 5 post-TNBS, corroborating MDS (Figure 1B). FSG-treated rats revealed a pronounced reduction in MD (p<0.05) compared with TNBS rats (Figure 1B). Colonic inflammation was also associated with increased mucosal neutrophil infiltration since colitic rats displayed an increase in MPO activity 24 h, 3 d and 5 d post-TNBS when compared to control non-inflamed rats (p<0.05). However, this increase was significantly higher (p<0.05) on day 3 post-TNBS *vs* 24 h and 5 d post-TNBS (Figure 1C). The oral treatment by FSG significantly reduced TNBS-induced increase in colonic MPO activity at all time points after induction of colitis (p<0.05) (Figure 1C). However, the effects of FSG treatment on MDS, MD and MPO activity did not reflect a total inhibition since values obtained for these parameters remained higher than in rats without colitis (Figure 1). All FSG effects on TNBS-induced colitis were blocked in the presence of the ER antagonist ICI 182.780 (Figure 1). Of note, FSG treatment in non-colitic rats had no effect *per se* on all inflammatory parameters evaluated compared with Ve-treated rats without TNBS colitis (Figure 1).

In a distinct series of experiments conducted in male rats, the preventive treatment with FSG decreased MPO activity at 24 h (p<0.05) and 3 d (p<0.01) post-TNBS when compared to corresponding Ve-treated colitic rats (see Figure S1). In addition, FSG given in a curative way in female rats (from TNBS administration until 5 days post-TNBS) decreased MPO activity on days 3 (p<0.01) and 5 (p<0.05) after induction of colitis (see Figure S2).

### FSG treatment affects cytokine profile in TNBS-colitic rats

**Figure 2:**
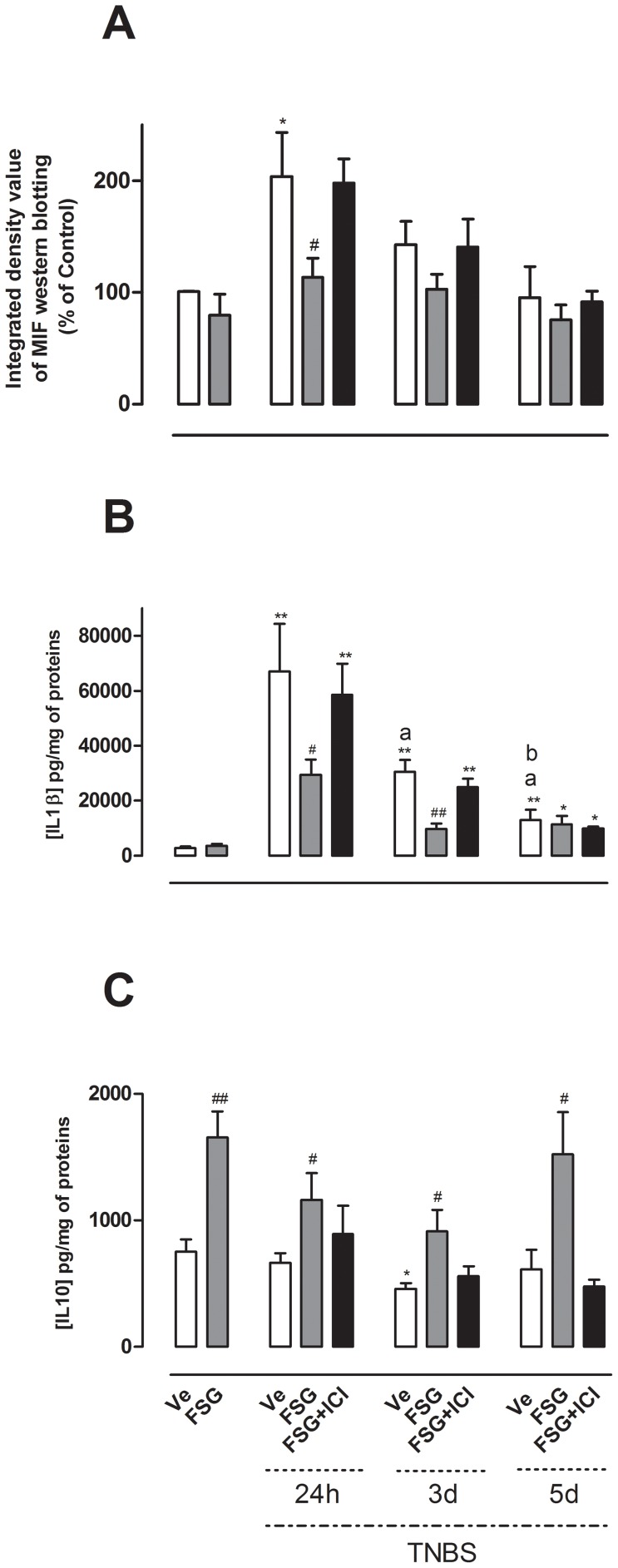
Effects of FSG treatment on cytokine profile after TNBS colitis. Effects of FSG on (A) MIF expression, (B) IL-1β, (C) IL-10 levels were evaluated in non inflamed colon and 24 h, 3 d, 5 d after TNBS-induced colitis in presence or absence of the ER antagonist ICI 182.780. Values are presented as means ± SEM. Statistical analysis were performed by One way-ANOVA with post hoc comparison by Tukey test. *p<0.05 *vs* non inflamed group, **p<0.01 *vs* non inflamed group, ^#^p<0.05 *vs* inflamed vehicle, ^##^p<0.01 *vs* inflamed vehicle, ^a^p<0.05 *vs* Ve-treated rats 24 h post-TNBS, ^b^p<0.05 *vs* Ve-treated rats 3 d post-TNBS.

In this set of experiments, we investigated the effects of oral treatment with FSG on the expression or release of the pro-inflammatory cytokines MIF and IL-1β, as well as the anti-inflammatory cytokine IL-10 in colonic tissues. MIF was significantly over-expressed only 24 h after TNBS administration (p<0.05) (Figure 2A) while colonic levels of IL-1β were markedly increased at 24 h, 3 d and 5 d after TNBS-induced colitis (p<0.01) (Figure 2B). FSG treatment did not affect colonic concentrations of MIF and IL-1β in non-colitic rats (Figures 2A, B). In contrast, the oral FSG treatment significantly decreased MIF expression 24 h after colitis (p<0.05) and IL-1β levels on days 1 and 3 post-TNBS (p<0.05; p<0.01 respectively) compared to corresponding values obtained in colitic rats (Figures 2A, B). The effects of FSG on MIF expression and IL-1β release were reversed in the presence of ICI 182.780 (Figures 2A, B). Interestingly, in non-colitic rats, as well as in colitic animals at 24 h, 3 d and 5 d post-TNBS, the FSG treatment significantly increased IL-10 release (p<0.05) (Figure 2C). The FSG-induced effects on IL-10 colonic levels were reversed following ICI 182.780 (Figure 2C).

### FSG prevents TNBS-induced increase in intestinal paracellular permeability

**Figure 3:**
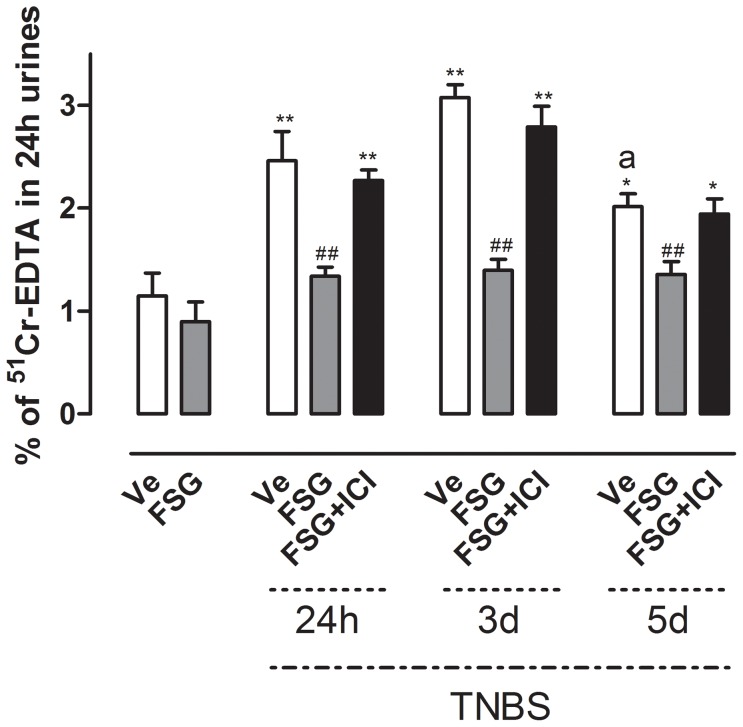
Effects of FSG treatment on TNBS-induced changes in intestinal paracellular permeability ± ER antagonist ICI182.780. Values are presented as means ± SEM. Statistical analysis were performed by One way-ANOVA with post hoc comparison by Tukey test. *p<0.05 *vs* non inflamed group, **p<0.01 *vs* non inflamed group, ^##^p<0.01 *vs* inflamed vehicle, ^a^p<0.05 *vs* Ve-treated rats 3 d post-TNBS.

Further, we assessed whether FSG treatment could improve TNBS-induced rise in intestinal permeability. TNBS administration significantly increased the IPP during the whole post-colitis period evaluated (Figure 3). This effect was more pronounced at 24 h and 3 d post-TNBS (p<0.01) than at 5 d (p<0.05). Moreover, no difference in TNBS-induced IPP changes was observed in colitic rats according to the phase of the sexual cycle (data no shown). FSG oral treatment strongly reduced the TNBS-induced increase of intestinal permeability (p<0.01) at all time points post-TNBS (Figure 3). The FSG effect on IPP in colitic rats was reversed by the ICI 182.780 administration (Figure 3).

### FSG prevents TNBS-induced visceral hypersensitivity

**Figure 4:**
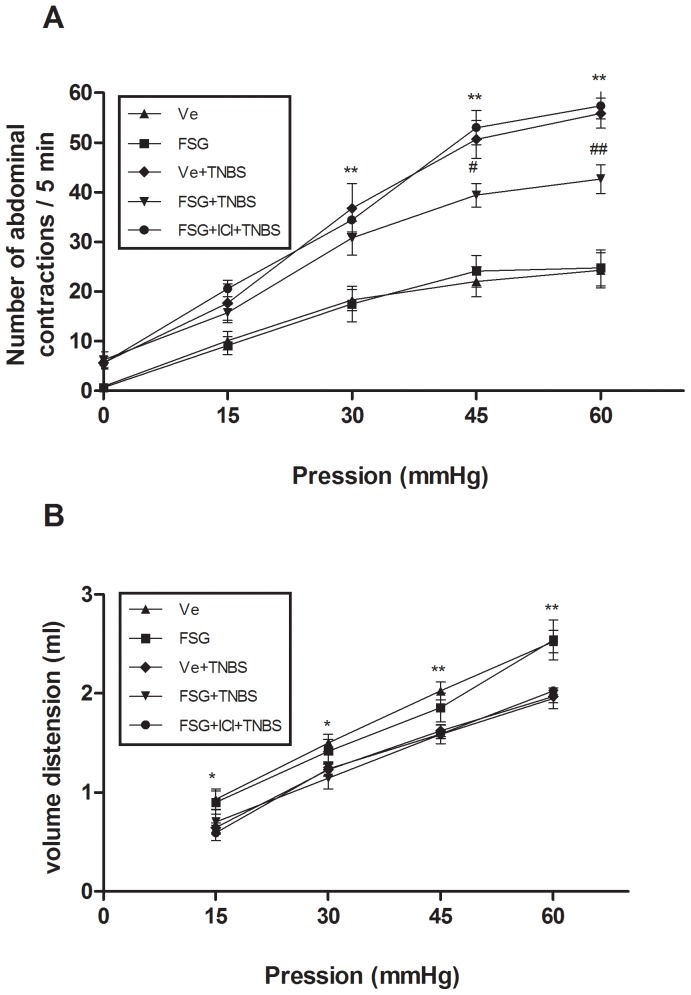
Effects of FSG treatment on (A) TNBS-induced visceral hypersensitivity in response CRD (B) colonic compliance. Abdominal response and colonic compliance were determined on 5^th^ day post-TNBS in presence or absence of the ER antagonist ICI 182.780. Values are presented as means ± SEM. Statistical analysis were performed by One way-ANOVA with post hoc comparison by Tukey test. *p<0.05 *vs* non inflamed group, **p<0.01 *vs* non inflamed group, ^#^p<0.05 *vs* inflamed vehicle, ^##^p<0.01 *vs* inflamed vehicle.

In a final set of experiments, we investigated the ability of FSG treatment to prevent the inflammation-associated visceral hypersensitivity. Five days after TNBS administration, the number of abdominal contractions at applied pressures of 30, 45 and 60 mmHg was significantly increased (p<0.01) in comparison with Ve-treated non-colitic group of rats (Figure 4A). FSG treatment strongly decreased (p<0.05) the TNBS-induced hypersensitivity to CRD (Figure 4A), while concomitant treatment with ICI 182.780 reversed this effect of FSG (Figure 4A). As expected, TNBS administration resulted in a decrease of colonic compliance (p<0.05) when compared to Ve-treated non colitic group (Figure 4B). FSG treatment did not affect colonic compliance in basal conditions (i.e. non-inflamed rats) in comparison with the Ve-treated non-colitic group (Figure 4B). Similarly, colonic compliance remained unchanged in FSG-treated *vs* Ve-treated colitic rats, indicating that the effect of FSG treatment on visceral sensitivity did not result from changes in muscle compliance.

### FSG treatment prevents TNBS-induced increase of faecal proteolytic activity and colonic PAR-2 over-expression

**Figure 5:**
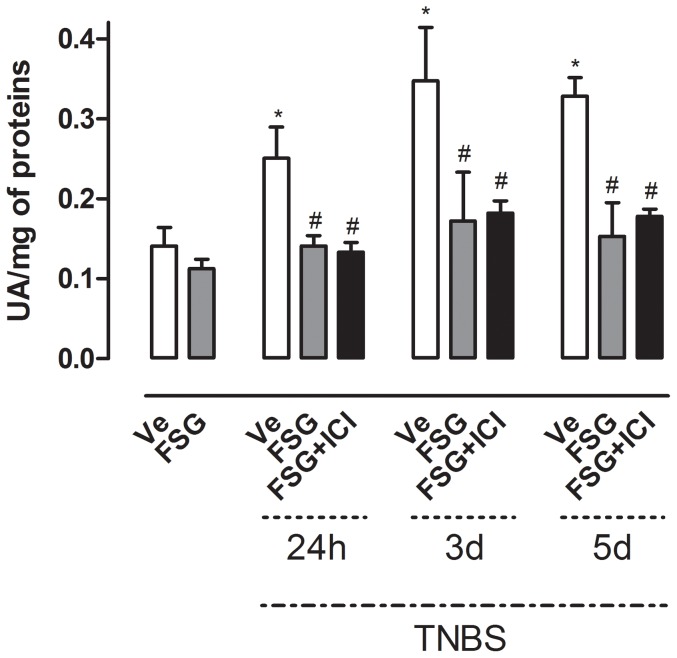
Effects of FSG treatment on TNBS-induced changes in faecal proteolytic activity ± ER antagonist ICI182.780. Data are expressed as means ± SEM. One way-ANOVA with post hoc comparison by Tukey test was used to analyse the data. *p<0.05 *vs* non inflamed group, ^#^p<0.01 *vs* inflamed vehicle.

**Figure 6:**
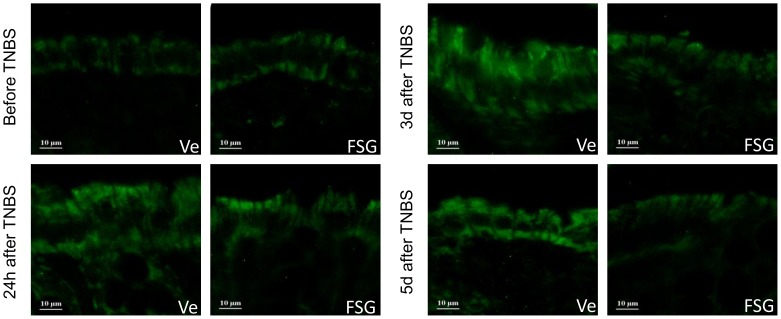
Immunohistochemical staining of longitudinal colonic section for PAR-2 expression. PAR-2 expression was evaluated in colonic epithelial cells in non inflamed colon and 24 h, 3 d, 5 d after TNBS-induced colitis in vehicle (Ve) or FSG treated rats.

Taking into account the BBI content of the FSG, we investigated its influence on intestinal proteolytic activity. Faecal proteolytic activity was significantly increased in colitic rats (p<0.05) from 24 h to 5 d post-TNBS (Figure 5). FSG treatment fully prevented this effect at all time-points post-TNBS (Figure 5). ICI 182.780 administration failed to reverse this FSG effect (Figure 5). Further, in basal conditions (i.e. non-colitic rats), a 15 d oral treatment by FSG did not affect faecal proteolytic activity since values obtained were similar to those obtained in Ve-treated non colitic rats (Figure 5). PAR-2 expression was increased in TNBS treated rats as reflected by immunochemistry from 24 h to 5 d post-TNBS (Figure 6). In basal conditions, FSG treatment did not affect PAR-2 expression in comparison to controls, whereas this treatment significantly reduced the TNBS-induced PAR-2 over-expression at all time-points post-TNBS evaluated (Figure 6).

## Discussion

Inflammatory bowel diseases, including CD and UC, are chronic inflammatory gastrointestinal conditions characterized by an inappropriate innate and adaptive immune systems response [2], increased luminal protease activity [6], over-expression of PAR-2 [27], epithelial barrier defects [3] and abdominal pain [1]. In this study, we show that an oral treatment with a fermented soy germ ingredient (FSG) reduces inflammatory response, intestinal permeability and visceral hypersensitivity in a rat model of colitis through two distinct pathways. These protective effects occurred partly through a phytoestrogen-derived ER-signalling in the gut, able to reduce pro-inflammatory cytokine profile and to enhance epithelial barrier function. Additionally, the protease inhibitor activity of the FSG compound prevented the increase of luminal protease activity, and decreased colonic epithelial PAR-2 over-expression in colitic rats, independently of ER-ligand activity. Overall, these preventive effects occurred with a daily-administered dose of isoflavones equivalent aglycone forms (0.45 mg per day/rat) close to the authorized isoflavone daily intake in humans according to the European safety guidelines (1 mg/kg BW/day).

TNBS intracolonically administered to rats induces immunologic responses and gut barrier defects reproducing symptoms characterising chronic and relapsing IBD conditions [28]. For example, pro-inflammatory cytokines including IL-1β, TNF-α or IL-6 are consistently elevated in IBD [29], and high serum levels of MIF are detected in patients with active CD [30]. MIF stimulates the T Helper (TH) 1 cytokine network (mainly IL-1β and TNF-α) involved in the acute inflammatory response and its maintenance [30], [31]. Similarly, these cytokines are produced in TNBS colitis [16]. In addition, transmural infiltration of neutrophils is a pathological feature of IBD [32]. TNBS colitis also increases MPO activity in the colonic mucosa, an index of neutrophil infiltration. Our study shows that FSG treatment prevented the colitis-induced increase in IL-1β and MIF in the rat colon. Concomitantly, the preventive FSG treatment prevented the increase in MPO activity in female and male rats, indicating inhibition of neutrophil influx in inflamed tissues. Since both MIF and IL-1β induce neutrophil recruitement [33], [34], and that blockade of MIF activity down-regulates IL-1β [31], [35], it is likely that the decrease of neutrophil influx and IL-1β release in the inflamed areas would result from FSG-mediated inhibition of MIF production in the acute phase of colitis. In addition, increased IL-10 limits TH1 immune responses [36], and deficiency in human IL-10 function related to IL-10 or its receptor IL-10R mutations was reported in the IBD pathogenesis [37]. FSG treatment increased basal IL-10 release and this effect was maintained in colitic rats. Since IL-10 production can inhibit pro-inflammatory cytokines release [38], we suggest that this mechanism also contributes to the preventive properties of FSG treatment, through downstream modulation of the TNBS-induced TH1 immune response. Interestingly, a reduction in the severity of TNBS colitis was also observed after curative treatment with FSG. Indeed, oral administration of FSG starting from the day of TNBS instillation to 5 days later resulted to a significant decrease in MPO activity on days 3 and 5 post-colitis (see Figure S2). This finding highlights that the anti-inflammatory effect exerted by the FSG treatment is mainly linked to early inflammatory cascade prevention in the colonic mucosa above reported in TNBS colitis.

In IBD as well as in experimental colitis models, oxidative stress has been incriminated in the inflammatory response. One may hypothesize that the antioxidant activity of FSG may contribute to alleviate TNBS injury. However, previous studies report that prevention of colonic inflammation through reduction of the reactive oxygen species (ROS) depends on the severity of colitis. Indeed, transgenic mice overproducing an antioxidant enzyme (CuZn-SOD) exhibit fewer lesions when submitted to mild, but not severe colitis [39]. The TNBS colitis used in the current study is considered as severe regarding the massive mucosal insult observed, mimicking acute IBD. In a study comparing the protective effects of different flavonoids against photo-oxidative stress, isoflavones demonstrate amplification of the photodamage, probably linked to their weak ability to scavenge ROS [40]. This is in agreement with previous literature showing that the structure of isoflavones confers weaker antioxidant properties when compared with other flavonoids [41]. Taken together, these data suggest that the anti-inflammatory effect of the FSG treatment reported herein is unlikely to be linked to its antioxidant properties.

Several studies report that estrogens have anti-inflammatory activity in colitis [16], [17], [42], partly occurring through ER-dependent down-regulation of MIF production by epithelial cells and macrophages [16]. ERs are widely expressed in the gut with marked expression of ERβ in colonic epithelial cells [43]. An ERβ agonist, ERB-041 displayed anti-inflammatory activity in the HLA-B27 transgenic rat model of IBD [42]. Of note, isoflavones such as genistein and daidzein act primarily through ERβ [14], and the aglycone forms are the gut absorbable forms [21]. Interestingly, the FSG compound tested, contains 85% of these phyotestrogens present in aglycone forms. Oral genistein displayed curative properties in TNBS colitic rats [10]. In this study, we show that the FSG anti-inflammatory effects as well as the increase on basal IL-10 release were blocked by the ER antagonist ICI 182.780, demonstrating that these properties are linked to ER-ligand activity.

Intestinal paracellular permeability (IPP) is increased in TNBS colitic rats, a situation that mimics epithelial barrier defect in IBD [3], [44]. IPP is mainly regulated by tight junction (TJ) proteins [45]. In IBD, the release of pro-inflammatory cytokines increases intestinal permeability [46], [47], partly through reduced expression of TJ proteins and/or disassembly of TJ protein complex [48]. We found that the FSG treatment limits the TNBS-induced increase of gut permeability, and this effect was blocked by ICI 182.780, highlighting involvement of an ER-ligand signalling pathway. These data are consistent with studies showing a protective role of isoflavones on intestinal TJ barrier [18]. ERβ signalling pathways in the colon decrease intestinal permeability through up-regulation of TJ protein expression, *in vivo* and in *vitro* [15]. Interestingly, estrogenic activity of FSG has been shown to counteract increased gut permeability by enhancing TJ protein expression in a stress rat model [22]. On the other hand, down-regulation of IL-10 contributes to increased intestinal permeability in colitis [49]. We suggest that FSG may enhance junctional complexes leading to prevention of IPP.

In addition to estrogenic properties, FSG contains BBI (a highly heat stable protease inhibitors) that may contribute to the anti-inflammatory activity. This characteristic (which is absent in phytoestrogen-enriched diets or a simple mixture of isolated isoflavones) is of importance since BBI have been reported to inhibit activated tissue proteases in colitic rats [50], and a concentrate of BBI exerts beneficial effects in UC patients [13]. In UC patients, increased faecal serine protease and colonic PAR-2 expression have been reported [27]. Herein, we show for the first time that TNBS exacerbates the PAR-2 expression in the rat colon during the whole post-TNBS period. This is in agreement with previous animal studies showing that PAR-2 deficiency protects from the development of inflammation induced by three different models of colitis, including TNBS [51]. In our study, we report that anti-inflammatory properties of FSG are also linked to blockade of PAR-2 over-expression in the inflamed rat colon, whereas this treatment did not change PAR-2 expression in basal conditions. Since PAR-2 activation by PAR agonists leads to increased intestinal permeability in rodents [4], we suggest that the FSG ability to inhibit protease activity and PAR-2 expression pattern in colitic rats contributes in synergy with the ER-ligand activity pathway to its anti-inflammatory properties.

Abdominal pain and spasms are common symptoms in IBD. In UC patients as well as in experimental colitis, rectal hypersensitivity and lowering of pain thresholds have been observed [1], [23]. We show that FSG treatment prevents TNBS-induced hypersensitivity to CRD without changes in colonic compliance, indicating that this antinocipeptive effect did not result from changes in colonic muscular tone. Further, FSG treatment prevents TNBS-induced visceral hypersensitivity through an ER-ligand activity pathway since this effect was reversed by ICI 182.780. This result contrasts with previous data showing that in ovariectomized (OVX) colitic rats, estradiol replacement enhances visceral signal processing following colonic inflammation [52]. A possible explanation to this discrepancy may be attributed to (i) the hormonal status of animals, i.e OVX *vs* cyclic female rats used herein or (ii) the route of administration, i.e subcutaneous injection *vs* chronic oral treatment. For instance, chronic FSG or estradiol oral treatment in stressed cyclic rats prevents stress-induced hypersensitivity, suggesting that these compounds orally absorbed did not influence the already primed nociceptive pathway by endogenous sexual steroids [22]. In our study, the FSG-induced decrease in pain sensitivity may be linked to its anti-inflammatory properties as well as its ability to decrease TNBS-induced rise in intestinal permeability. Indeed, FSG treatment decreases IL-1β release in colitic rats, a cytokine able to sensitize the capsaicin receptor TRPV1 involved in inflammatory hyperalgesia [53]. Additionally, serine proteases such as cathepsins released during colitis, activate nociceptors to induce visceral pain via PAR-2 [54], and FSG may limit this pathway. Overall, the FSG-mediated improvement of epithelial barrier integrity restricts the passage of luminal agents, thus the release of pro-inflammatory mediators able to sensitize nerve afferences, thus leading to downstream visceral pain.

In conclusion, the anti-inflammatory properties of FSG treatment result from two distinct but synergic pathways linked to its composition. The phytoestrogenic property activates the ER-ligand activity related pathway, whereas the presence of BBI involves a PAR-2 mediated pathway. Highlighting these mechanisms of action provide rationale for potential use of this compound as adjuvant therapy in IBD.

## Supporting Information

Figure S1 **Effects of FSG treatment on the severity of TNBS-induced colitis in male rats.** Male Wistar rats were given orally for 15 d either vehicle or FSG treatment. Induction of TNBS colitis was performed on day 15 of the treatment as reported in females (see Materials and Methods section). Rats were sacrificed 24 h and 3 d post-colitis. Colon samples were taken for myeloperoxidase activity (MPO) assessments as described in Materials and Methods. Colonic MPO level showed significant neutrophil infiltration 24 h and 3 d post-TNBS in comparison to non-inflamed male rats (p<0.01). As observed in females (Figure 1C), FSG pretreatment resulted in decreased MPO activity (p<0.05) at 24 h and 3 d post-colitis compared to Ve-treated animals. Values are means ± SEM. ^**^p<0.01 *vs* basal, ^##^p<0.01, ^#^p<0.05 *vs* corresponding Ve group, ^a^p<0.05 *vs* Ve group of rats at 24 h post-TNBS.
(TIF)

Figure S2 **Curative effect of FSG treatment in female rats submitted to TNBS colitis.** Female Wistar rats were orally given daily either vehicle or FSG from the day of TNBS colonic instillation until sacrifice at 24 h, 3 d and 5 d post-colitis. TNBS administration induced a significant increase in MPO activity at 24 h, 3 d and 5 d post-colitis (p<0.01). Interestingly, a daily oral treatment with FSG beginning the day of TNBS administration induced a drop of colonic MPO levels at 3 and 5 d post-TNBS in comparison to Ve control rats (p<0.05). Values are means ± SEM. ^**^p<0.01 *vs* basal, ^##^p<0.01, ^#^p<0.05 *vs* corresponding Ve group, ^a^p<0.05 *vs* Ve group of rats at 3 d post-TNBS.
(TIF)
