# Supplementary figures and images for: A Low Dose of Fermented Soy Germ Alleviates Gut Barrier Injury, Hyperalgesia and Faecal Protease Activity in a Rat Model of Inflammatory Bowel Disease
Source: PLoS One. 2012 Nov 14;7(11):e49547. doi: 10.1371/journal.pone.0049547 (PMC3498131; doi:10.1371/journal.pone.0049547)

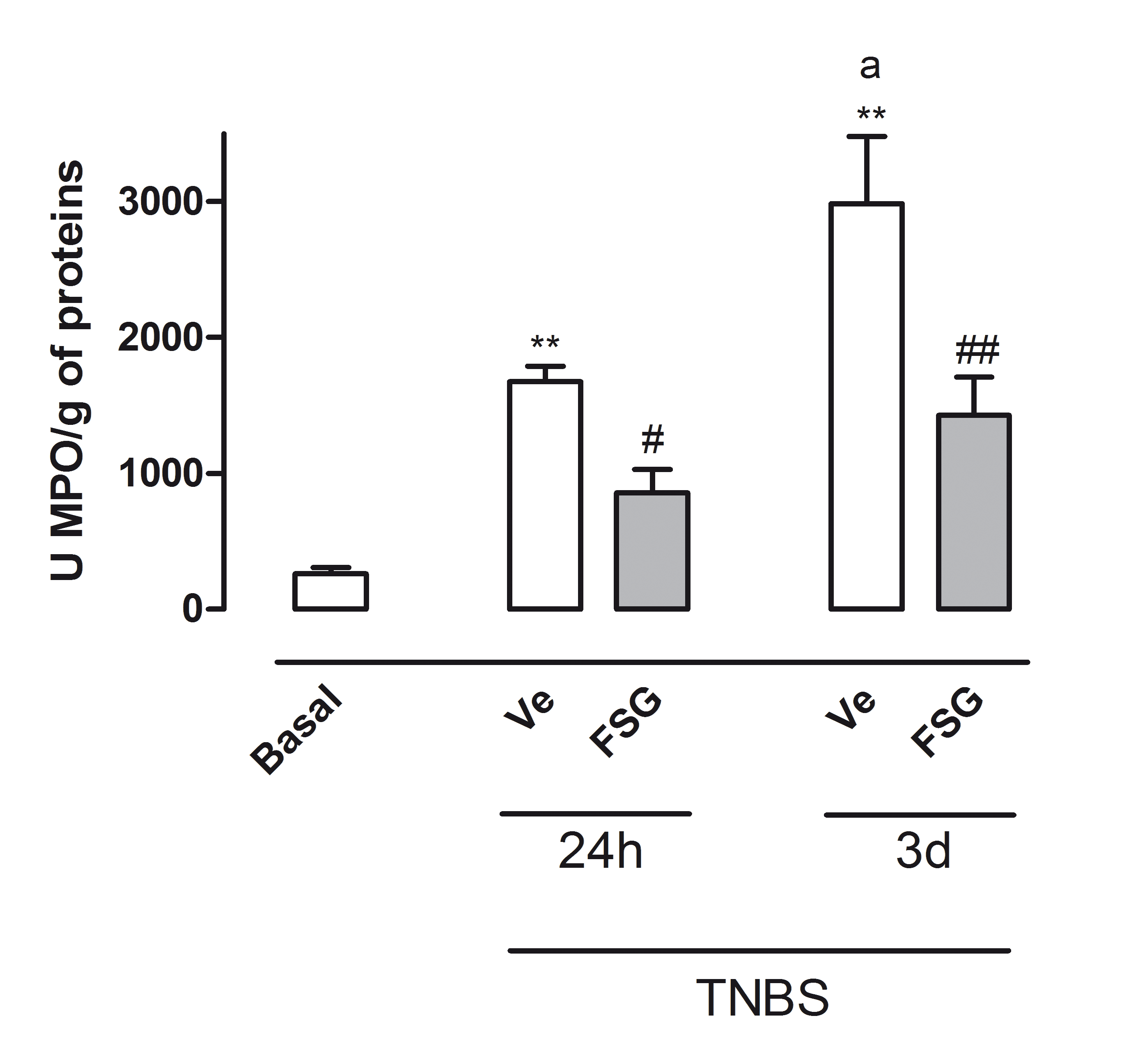

Supplement: Figure S1 — Effects of FSG treatment on the severity of TNBS-induced colitis in male rats. Male Wistar rats were given orally for 15 d either vehicle or FSG treatment. Induction of TNBS colitis was performed on day 15 of the treatment as reported in females (see Materials and Methods section). Rats were sacrificed 24 h and 3 d post-colitis. Colon samples were taken for myeloperoxidase activity (MPO) assessments as described in Materials and Methods. Colonic MPO level showed significant neutrophil infiltration 24 h and 3 d post-TNBS in comparison to non-inflamed male rats (p<0.01). As observed in females (Figure 1C), FSG pretreatment resulted in decreased MPO activity (p<0.05) at 24 h and 3 d post-colitis compared to Ve-treated animals. Values are means ± SEM. **p<0.01 vs basal, ##p<0.01, #p<0.05 vs corresponding Ve group, ap<0.05 vs Ve group of rats at 24 h post-TNBS. (TIF) [file pone.0049547.s001.tif]

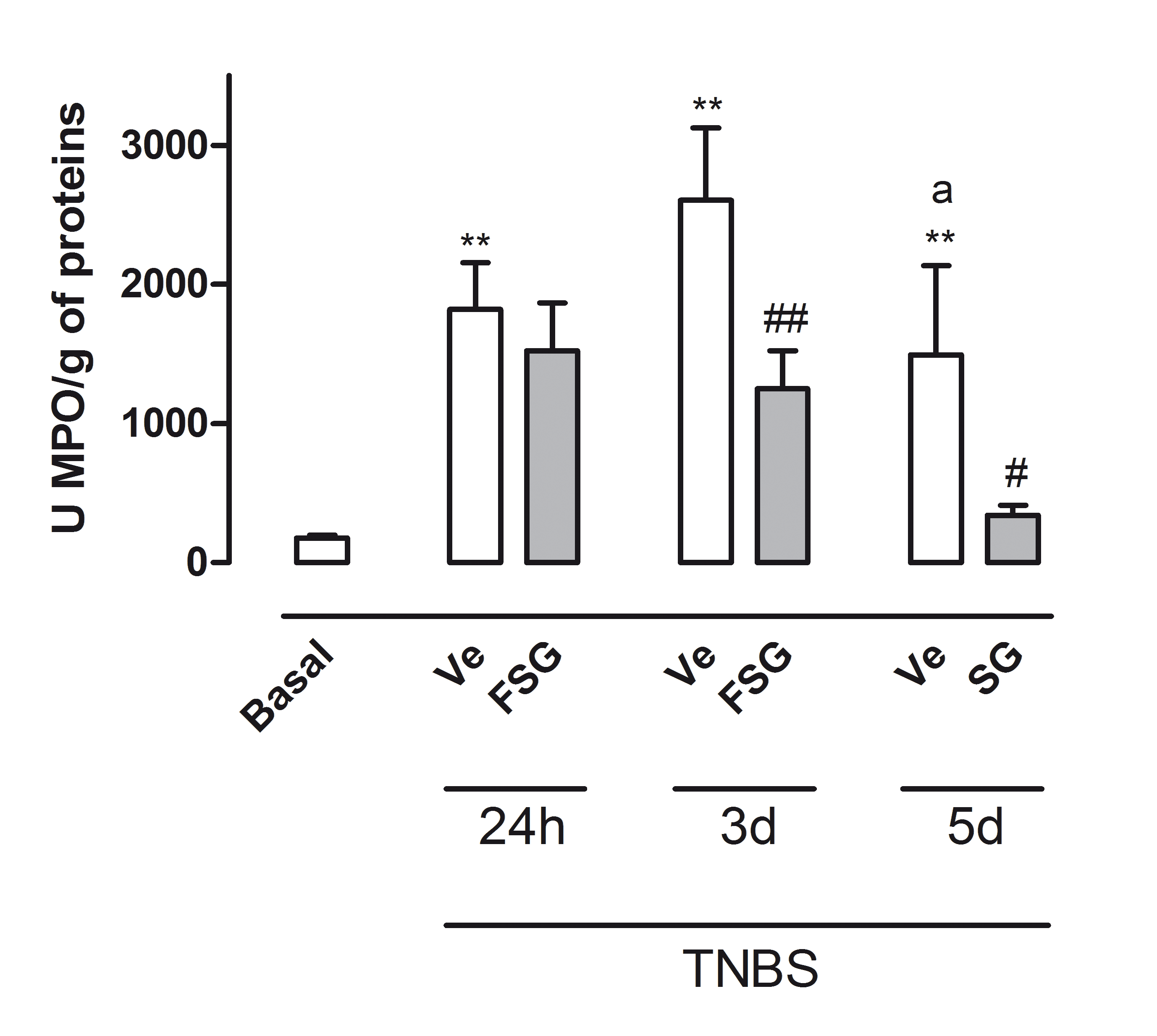

Supplement: Figure S2 — Curative effect of FSG treatment in female rats submitted to TNBS colitis. Female Wistar rats were orally given daily either vehicle or FSG from the day of TNBS colonic instillation until sacrifice at 24 h, 3 d and 5 d post-colitis. TNBS administration induced a significant increase in MPO activity at 24 h, 3 d and 5 d post-colitis (p<0.01). Interestingly, a daily oral treatment with FSG beginning the day of TNBS administration induced a drop of colonic MPO levels at 3 and 5 d post-TNBS in comparison to Ve control rats (p<0.05). Values are means ± SEM. **p<0.01 vs basal, ##p<0.01, #p<0.05 vs corresponding Ve group, ap<0.05 vs Ve group of rats at 3 d post-TNBS. (TIF) [file pone.0049547.s002.tif]
